# Predicting Glycemic Index and Glycemic Load from Macronutrients to Accelerate Development of Foods and Beverages with Lower Glucose Responses

**DOI:** 10.3390/nu11051172

**Published:** 2019-05-25

**Authors:** Andreas Rytz, Dorothée Adeline, Kim-Anne Lê, Denise Tan, Lisa Lamothe, Olivier Roger, Katherine Macé

**Affiliations:** 1 Nestlé Research Center, 1000 Lausanne, Switzerland; kim-anne.le@rdls.nestle.com (K.-A.L.); lisa.lamothe@rdls.nestle.com (L.L.); olivier.roger@rdls.nestle.com (O.R.); catherine.mace@rdls.nestle.com (K.M.); 2 Science and Technology, CPW, 1350 Orbe, Switzerland; dorothee.adeline@rd.nestle.com; 3 Nestlé R&D Center, Singapore 618802, Singapore; denise.tan@rdsg.nestle.com

**Keywords:** glycemic index, glycemic load, macronutrient composition, model

## Abstract

Low glycemic index (GI) and/or low glycemic load (GL) are associated with decreased risks of type-2 diabetes and cardiovascular disease. It is therefore relevant to consider GI and GL in the early phases of the development of packaged foods and beverages. This paper proposes a model that predicts GI and GL from macronutrient composition, by quantifying both the impact of glycemic carbohydrates and the GI-lowering effects of nutrients such as proteins, fats and fibers. The precision of the model is illustrated using data on 42 breakfast cereals. The predictions of GI (*r* = 0.90, median residual = 2.0) and GL (*r* = 0.96, median residual = 0.40 g) compete well with the precision of the underlying in-vivo data (Standard Error SE = 3.5 for GI). This model can guide product development towards lowering GI and GL, before final confirmation by in vivo testing.

## 1. Introduction

Carbohydrates should account for 50% of total energy intake in a normal diet, but free sugars should account for less than 10%, as recommended by the World Health Organization, to prevent both obesity and dental caries [1]. In order to achieve this public health target, consumers will need to make significant changes to their diets and providers of packaged food and beverages will need to replace free sugars with alternatives that are truly supportive of health benefits. Sugars have many functionalities that go beyond providing sweetness. Therefore, complete removal or partial reduction of sugar is not simple and various routes for nutritious sugar replacements have been proposed [2]. The first approach consists of carefully selecting alternative glycemic carbohydrates in order to avoid replacing low glycemic sugars such as lactose by high glycemic carbohydrates such as maltodextrin. The second approach consists in replacing sugars and rapidly digestible glycemic carbohydrates by slowly digestible carbohydrates [3], non-glycemic carbohydrates such as dietary fibers [4], and particularly β-glucans [5]. Further considering non-glycemic nutrients such as proteins and fats is important since they further modulate postprandial glycemic response [6,7,8].

Foods and beverages with low glycemic index (GI) and low glycemic load (GL) are considered beneficial [9], based on evidence coming from meta-analyses of both observational studies [10,11], as well as from randomized clinical trials [12]. Benefits include the management of diabetes [13], the prevention of diseases such as type-2 diabetes [14], and cardiovascular disease, including coronary heart disease [15,16].

To estimate the GI/GL of a meal or a diet, one can use the model of the Food and Agriculture Organization (FAO) [17]. This model predicts the GI of a diet as a simple function of its constituting products by using average GI and GL values of hundreds of common foods and beverages available in generic tables [18]. This simple model is unfortunately not always reliable; for example, it failed to predict GI of composite breakfast meals [19,20]. The FAO-model has two inherent limitations. First, the GIs of the constituting products are averages coming from tables that do not necessarily reflect the specificities of the actual products (e.g., depending on the local sources of ingredients and processes, the reported GIs of white bread made from wheat flour varies between 59 and 89). Second, the model does not properly take into account the GI-lowering effect of the non-glycemic components in the meal (e.g., the butter on the bread).

This work presents a new model that builds on the logic of the FAO-model, but that overcomes its two inherent limitations. First, in order to overcome the issue of product specificities, and in order to be useful for both complete meals and single products, it models GI as a function of nutrients, not as a function of products (with GI = 100 for pure glucose). Second, it both deterministically quantifies the effects of glycemic carbohydrates and empirically estimates the GI-lowering effects of other macronutrients, such as proteins, fats and fibers. 

Published data, including data on honey, pasta, bread and milk, allowed us to set up the shape of the model combining deterministic and empirical aspects. Sixty in-house in-vivo trials allowed the estimation of empirical coefficients of the model. These 60 trials covered various product categories (i.e., infant cereals, cereal bars, biscuits and dairy beverages) and a wide range of recipes, leading to GI spreading between 15 and 95. Consequently, the correlation between prediction and observation was very large (*r* = 0.97, *p*-value < 0.01, *n* = 60). In order to challenge the model, it has been applied on a single product category that was not part of the model setup. The application presented includes correlation plots for 42 breakfast cereals, as well as the corresponding Bland-Altman difference plots [21].

## 2. Materials and Methods

### 2.1. Products

Forty-two breakfast cereals made from various grains (i.e., wheat, oat, mixtures of grains), with or without inclusions (e.g., chocolate, fruits, nuts, honey), have been characterized for their macronutrient composition using standard analytical methods [22]. The type of process and composition of these 42 starch-based products is presented (Table 1). These products contained glycemic carbohydrates accounting for 57 to 82 g/100 g and the proportion of Rapidly Digestible Starch ranged between 65% for granola and 88% for extruded products.

### 2.2. In-Vivo Testing

The in-vivo testing to measure GI was performed according to international standards [23]. Fourteen studies, all approved by the Human Research Ethics Committee of the University of Sydney, tested the 42 products with 2 to 5 products per study protocol. The constant test protocol enabled the combination of data from all 14 studies to perform secondary analyses. Serving sizes were calculated to deliver 50 g glycemic carbohydrates. Test products were compared with a reference of 50 g glucose, which was assessed in triplicate in each study. Within each study, 10 healthy subjects consumed all test products and the reference in a crossover design, with one sample per day, under fasting conditions, with at least one-day washout between two test days. Subjects tested the dry products along with a glass of 250 mL water. Two fasting blood samples (t = −5 min and t = 0 min) were obtained, and after the second fasting blood sample a test product was consumed and postprandial glycaemia was monitored for 120 min (15, 30, 45, 60, 90, 120 min). A total of 140 subjects (61 women, 79 men) were enrolled in these 14 studies with age ranging between 19.8 and 52.3 years (mean = 29.6, SD = 8.6) and BMI ranging between 18.2 and 24.9 kg/m^2^ (mean = 22.0, SD = 2.2). GI and GL (Equations (1a) and (1b)) were determined using commonly accepted equations [24]:

(1a) $$\mathrm{GI}=\frac{\mathrm{Incremental}\;\mathrm{blood}\;\mathrm{glucose}\;\mathrm{area}\;\mathrm{of}\;\mathrm{the}\;\mathrm{test}\;\mathrm{food}}{\mathrm{Incremental}\;\mathrm{blood}\;\mathrm{glucose}\;\mathrm{area}\;\mathrm{of}\;\mathrm{glucose}}\times 100$$

(1b) $$\mathrm{GL}=\frac{\mathrm{GI}\;x\;\mathrm{glycemic}\;\mathrm{carbohydrate}\;\mathrm{per}\;\mathrm{serving}\;\left(g\right)}{100}$$

### 2.3. FAO Predictive Model

The FAO-model predicts GI of a meal as a function of its N constituting foods. If c_i_ is the amount (in grams) of glycemic carbohydrates of the i^th^ food (i = 1… N) and GI_i_ its GI, the model can be written in a synthetic form (Equation (2)):

(2) $$GI_{Meal}=\frac{\sum_{i=1}^{N}c_{i}GI_{i}}{\sum_{i=1}^{N}c_{i}}$$

As an example, using published data [25], consider a simple test meal composed of only two foods, namely 110 g white bread (c_1_ = 50 g glycemic carbohydrates, GI_1_ = 88) and 30 g butter (c_2_ = 0g, GI_2_ = 0). The equation predicts GI_Meal_ = (50 × 88 + 0 × 0)/(50 + 0) = 88, whereas the reported in-vivo data suggest that the addition of butter reduces GI of the white bread from 88 to 67. This example illustrates the inability of the current model to account for the GI-lowering effect of fat (and similarly for proteins or fibers).

### 2.4. Development of a New Model to Predict GI

The main idea of the new model is to apply the logic of the FAO-model on macronutrients instead of foods. The model development includes three steps (Equations (3a)–(3c)).

In a first step, the model considers products composed exclusively of water and of N mono/disaccharides. Let x_i_ be the relative amount (%) of the i^th^ mono/disaccharide (i = 1… N) and GI_i_ its tabulated GI (Table 2), given as an average of GI-values reported by the University of Sydney [26]. Sugar alcohols that are partially glycemic such as maltitol (GI = 35) or xylitol (GI = 12) should be considered as mono/disaccharides in this context. With these definitions, the synthetic form of the FAO-model remains unchanged (Equation (3a)), while accurately predicting the GI of any mono/disaccharide mix.

(3a) $$GI=\frac{\sum_{i=1}^{N}x_{i}GI_{i}}{\sum_{i=1}^{N}x_{i}}$$

As a fictive example, a syrup composed of 90 g/100 g water and a mix of 3 g glucose (GI = 100), 2 g fructose (GI = 20) and 5 g maltitol (GI = 35) would yield GI = (3 × 100 + 2 × 20 + 5 × 35)/(3 + 2 + 5) = 51. In-vivo data of such mixes are hardly available, but various published data on products such as honey tend to confirm the accuracy of the model [27].

In a second step, the model extends mono/disaccharides to glycemic carbohydrates, including glycemic polysaccharides. Equation (3a) still properly handles polysaccharides such as maltotriose, maltotetraose or more generally maltodextrin of any dextrose equivalent, but not starch. GI of starch varies due to botanical diversity and the fact that processing can affect the availability of glucose as demonstrated by the impact on GI of various ways of cooking pasta [28], or of cooling cooked rice [29]. Equation (3b) accounts for this change in starch availability by introducing a correcting factor a_i_ to characterize the availability of the glycemic carbohydrates.

(3b) $$GI=\frac{\sum_{i=1}^{N}x_{i}a_{i}GI_{i}}{\sum_{i=1}^{N}x_{i}}$$

As shown in Table 2, a_i_ = 1 for all glycemic carbohydrates except for starch for which it is best estimated by the proportion (0 ≤ a_i_ ≤ 1) of Rapidly Digestible Starch [30].

In the third step, the model quantifies the impact on GI of all non-glycemic nutrients. Any product can be considered as composed of N nutrients (N = m + n) with m glycemic carbohydrates and n other nutrients, with x_k_ the relative amount (%) of the k^th^ nutrient (k = 1… N) and ∑x_k_ = 100%. Equation (3c) introduces the non-glycemic nutrients as diluting factors of GI, along with coefficients b_j_ (j = 1… n) that characterizes their GI-lowering power.

(3c) $$GI=\frac{\sum_{i=1}^{m}x_{i}a_{i}GI_{i}}{\sum_{i=1}^{m}x_{i}+\sum_{j=1}^{n}x_{j}b_{j}}$$

Non-glycemic nutrients without GI-lowering power (e.g., water) have b_j_ = 0, whereas b_j_ of other nutrients were a-priori unknown and were estimated to be highest for β-glucans, fats and proteins (0.6), followed by other soluble fibers (0.3) insoluble fibers and ashes (Table 3). These empirical coefficients were estimated by Ordinary Least Square (OLS) regression applied on a heterogeneous dataset of 60 in-vivo studies, not including the 42 breakfast cereals (data not shown).

As an example, published data point to a GI of 66 for a test product composed of 50% glucose and 50% proteins [31]. This is close to the GI as predicted by Equation (3c) with GI = 50% × 100/(50% + 50% × 0.6) = 63. Other published data report GI = 27 for 100 g of whole milk composed of 88 g water, 4.9 g lactose, 3.3 g fat, 3.1 g protein and 0.7 g minerals [32]. In comparison, Equation (3c) yields a predicted GI of 26 with GI = 4.9 × 47/(4.9 + 88 × 0 + 3.3 × 0.6 + 3.1 × 0.6 + 0.7 × 0.0) = 26. Finally, coming back to the example of the 30 g of butter on 110 g white bread, Equation (3c) predicts a decrease from GI = 88 for plain bread to GI = 65 for bread + butter (vs. GI = 67 in-vivo). Similar decreases have been observed when replacing dairy butter with peanut butter [33].

Predicted glycemic load (GL) is derived from predicted GI using the standard GL Equation (1b).

## 3. Results and Discussion

### 3.1. Precision of Predictions for 42 Breakfast Cereals

**Figure 1:**
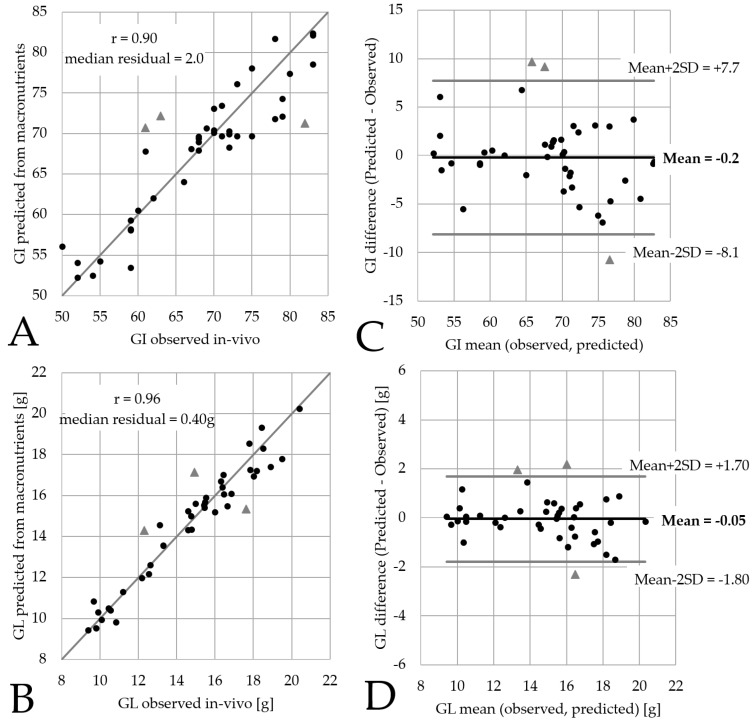
Macronutrient predictions vs. in-vivo data for 42 breakfast cereals with (**A**) observed vs. predicted GI, (**B**) observed vs. predicted GL, (**C**) GI Bland-Altman plot (**D**) GL Bland-Altman plot

In-vivo GI of 42 breakfast cereals ranged between 50 and 83 (mean = 68, SD = 9.2). The granola and muesli products tested had the lowest GI (50–60), before bars (66–72), whereas flakes and extruded products cover a wider range depending on their composition (61–83). The standard error (SE) of in-vivo data is SE = 3.5. Predicted GI of the same breakfast cereals ranged between 52 and 82 (mean = 68, SD = 8.2). When visualizing observed vs. predicted GI (Figure 1A), the correlation is *r* = 0.90 (*p*-value < 0.01) and the precision of the prediction given by the median absolute residual (=2.0) is smaller than the in-vivo SE.

With a standard serving size of 30 grams of product, the in-vivo GL of these 42 breakfast cereals ranged between 9.4 and 20.4 grams (g) of glucose equivalent (mean = 14.7 g, SD = 3.1 g) and their predicted GL range between 9.4 g and 20.2 g (mean = 14.7 g, SD = 2.9 g). When visualizing observed vs. predicted GL (Figure 1B), the correlation is *r* = 0.96 (*p*-value < 0.01) and the precision of the prediction was high since the median absolute residual was as low as 0.40 g. 

Three products featuring specific inclusions (i.e., cashew nuts, almonds, quinoa or cocoa) yielded predictions differing from actual in-vivo data by 9–11 points for GI and by 2.0–2.3 g for GL. These three products are also outside the mean ± 2SD range in the Bland-Altman difference plots (Figure 1C,D). These plots show that the predictions were not biased and that no simple relationships between the differences and the mean value can be identified. 

These results illustrate the strength of the new model that combines deterministic modelling to quantify the impact of glycemic nutrients and empirical modelling to quantify the impact of GI-lowering nutrients. This hybrid model delivers more accurate predictions than pure empirical models such as the one described by Meynier [34], who used advanced feature selection techniques to select main effects and interactions of four product characteristics (slowly digested starch, fat, fiber, and rapidly digested starch) to predict the GI of cereal products (*r* = 0.73, *p*-value < 0.01).

The proposed model delivers accurate GI and GL prediction for products with high proportion of glycemic nutrients such as the 42 tested breakfast cereals. This is because it first captures the effect of glycemic nutrients in a very simple deterministic way by modelling the GI of a mix of glycemic nutrients as the weighted average of their GIs and second accounts for the GI-lowering effect of other nutrients. Glycemic nutrients include mono/disaccharides and glycemic polysaccharides as well as nutrients such as maltitol and xylitol, which are partially glycemic, and therefore increase glycemic response. Non-glycemic nutrients include fat, fibres, proteins, ashes and water. The provided list of nutrients is not exhaustive and can be adapted to any product category, including novel glycemic or non-glycemic nutrients if needed.

### 3.2. Limitations of the Model

Whilst the deliberate simplicity of the model facilitates user experience, it does limit its accuracy. As a case in point, among the 42 breakfast cereals tested, the predicted GL was too high for two products with inclusions of cashew nuts, almonds and quinoa and too low for a product rich in cocoa. These products reveal that the model is not fully capturing the specificities of components that are rich in GI-modulating nutrients such as particular fat types or polyphenols [35].

More generally, a limitation of the model is that starch digestibility is captured through a single global marker (i.e., coefficient a_i_) regardless of the underlying mechanisms. This global marker is well estimated by the fraction of rapidly digestible starch, as suggested by the empirical data used to build the model. But this simple approach is eventually not properly addressing mechanisms such as the modulation of enzyme availability induced by specific ingredients (e.g., quinoa, lentils or chickpeas), the impact of ingredient processing on particle sizes within the same grain (e.g., cracked wheat vs. wheat flour), the impact of process on products comparable in composition (e.g., bread vs. pasta) [36], and the complex interactions occurring among food constituents, especially when the physical format of the product varies (e.g., solid vs. liquid) [37]. Such mechanisms might be captured more adequately by alternative in-vitro methodologies that have shown to be highly correlated with in-vivo GI [38,39]; but such methodologies rely on long and tedious procedures that conflict with the simplicity of the proposed model.

Another potential limitation of the model is that it captures the GI-lowering effect of non-glycemic nutrients in the same way regardless of the underlying mechanism. This simplifies the tool, but limits the accuracy for estimating the GI-lowering power of various nutrients. For example, proteins—similarly to fibers and fats—might increase viscosity and therefore physically protect glycemic nutrients from enzymatic digestion; but in addition, some amino acids are known to trigger the insulin response and therefore to lower the glycemic response [40]. Consequently, the b-coefficient of proteins in the model might strongly vary according to their amino acid profiles leading to two or more protein categories (e.g., low and high insulinogenic effect). 

### 3.3. Potential Use of Model Beyond Breakfast Cereals

The estimated b-coefficients should be adapted for each product category based on empirical data. As an example, the model was initially developed with total fibers instead of a split into three fiber types (insoluble, soluble and β-glucans). It worked well on tested products that featured fibers of similar type (i.e., constant ratio of soluble to insoluble, no β-glucans), however, the method became inaccurate when comparing products containing a variety of fibers. Splitting total fibers into three nutrients indirectly integrates the modelling of viscosity [41]. This logic could be extended to further splitting β-glucans (and including other fibers such as pectin, glucomannans or acacia gum) according to their effect on viscosity [42], or to their molecular weight [43]. Similarly, the model could be extended to split fats into low and high viscous fats [44], or according to their impact on gastric emptying.

The proposed model is scalable and should theoretically become more precise with more detailed nutrient breakdowns. However, the more detailed the input composition, the more difficult it becomes to access this information in a precise manner. Consequently, there is a trade-off for each product category and each application of the model. As an example, the model used back of pack labelling to benchmark prepared composite meals, such as pizzas, enchiladas or burritos. The GI predictions were relevant since structural and microstructural transformations happening during processing, as well as resulting starch availability were well mastered at factory level and the reheating by consumers was expected to have minimal additional impact. In this sense, the model might be more relevant for finished packaged foods than for semi-finished foods (e.g., pizza dough) or homemade meals for which cooking procedures can have a strong impact. The benchmark of prepared composite meals helped to prioritize brands to be improved. Once these products are identified, product developers could systematically vary their processes and ingredient parameters in order to identify settings that minimize estimated GI and GL, whist achieving the ideal sensory profile [45].

## 4. Conclusions

This work presents a model that provides precise analytical predictions of GI and GL in the case of breakfast cereals. It quantifies both the impact of glycemic nutrients and the GI-lowering effect of other macronutrients. Limitations of the model and potential usage of the model for other product categories are discussed. These analytical predictions are more precise for GL than for GI, which is interesting because GL is a proxy of the physiological glycemic response, taking into account not only the characteristics of macronutrients but also their quantity. The model is therefore particularly useful for products for which the mode of preparation can vary. Indeed, breakfast cereals can be consumed dry or reconstituted with milk (whole, semi-skimmed or skimmed), with or without further additions (e.g., added sucrose). As an example, when adding 125 mL of whole milk on top of the 30 g of cereals, the GL of the 42 breakfast cereals tested was predicted to increase between 0.0 to 1.2 g (mean = 0.56 g, SD = 0.32). The resulting GL is below the simple addition of the two GLs of cereals and whole milk (i.e., +1.7 g for 125 mL), simply because milk protein and milk fat act as GI-lowering agents for the glycemic carbohydrates of the cereals. In such cases, the model allows estimation of GI and GL for any mode of preparation. The model can therefore help the food industry to accelerate the development of breakfast cereals (and potentially other foods and beverages) with lower glucose responses, while taking into account personalized usages and modes of preparation. Prototypes with the highest predicted potential should then be tested in-vivo to confirm the physiological accuracy of these statistical predictions.

## Figures and Tables

**Table 1:** 42 breakfast cereals with process, composition [g/100 g], glycemic index (GI) (in-vivo vs. predicted) and glycemic load (GL) [g] for a 30 g serving (in vivo vs. predicted), sorted by descending GI.

	Process	Sucrose (g/100)	Starch (g/100)	Fiber Sol. (g/100)	Fiber Ins. (g/100)	Fat (g/100)	Protein (g/100)	GI	GI pred.	GL (g)	GL pred. (g)
P01	Extruded	11.8	61.2	2.4	7.1	2.2	9.1	83	79	18.2	17.2
P02	Extruded	10.9	71.0	0.7	2.0	2.1	6.9	83	82	20.4	20.2
P03	Extruded	6.3	68.0	1.9	5.8	3.3	8.3	83	82	18.5	18.3
P04	Flakes	14.9	56.8	2.8	8.3	2.2	10.3	82	71	17.6	15.3
P05	Extruded	16.8	57.5	2.2	6.7	2.1	8.5	80	77	17.8	17.3
P06	Flakes	11.8	64.2	1.7	5.1	1.4	9.3	79	74	18.0	16.9
P07	Flakes	28.6	53.7	0.8	2.3	2.5	6.3	79	72	19.5	17.8
P08	Extruded	4.1	74.7	0.7	2.0	1.4	12.3	78	82	18.4	19.3
P09	Extruded	28.8	52.0	1.1	3.3	2.7	6.3	78	72	18.9	17.4
P10	Extruded	4.0	62.6	3.2	9.7	2.1	12.3	75	78	15.0	15.6
P11	Extruded	25.2	48.8	2.2	6.5	4.0	7.8	75	70	16.7	15.5
P12	Extruded	30.0	46.9	1.5	4.4	4.8	6.2	73	70	16.8	16.1
P13	Flakes	11.4	69.8	1.0	2.9	2.1	7.1	73	76	17.8	18.5
P14	Bar	27.4	41.0	1.4	4.1	7.1	6.3	72	70	14.8	14.3
P15	Extruded	29.8	44.3	1.9	5.7	3.9	8.4	72	68	16.0	15.2
P16	Extruded	25.0	51.2	1.6	4.8	5.0	7.2	72	70	16.5	16.1
P17	Bar	27.9	40.6	1.5	4.5	7.3	6.2	71	70	14.6	14.3
P18	Flakes	17.2	60.0	1.6	4.7	1.2	8.6	71	73	16.4	17.0
P19	Bar	21.3	48.2	1.3	3.8	6.5	5.8	70	73	14.6	15.2
P20	Extruded	29.7	48.3	1.5	4.6	3.0	7.6	70	70	16.4	16.4
P21	Extruded	20.9	52.6	1.9	5.8	3.7	8.8	70	70	15.4	15.5
P22	Extruded	29.8	49.0	1.3	4.0	5.0	4.9	69	71	16.3	16.7
P23	Bar	22.5	42.7	1.4	4.3	9.8	6.3	68	69	13.3	13.6
P24	Extruded	28.7	47.0	1.4	4.1	7.4	7.6	68	68	15.4	15.4
P25	Extruded	28.8	47.0	1.6	4.7	4.5	8.0	68	69	15.5	15.7
P26	Flakes	28.4	47.7	2.2	6.6	1.7	8.7	68	70	15.5	15.9
P27	Extruded	24.8	48.6	1.3	3.9	10.1	5.7	67	68	14.8	15.0
P28	Bar	30.0	33.4	1.0	3.0	12.9	7.4	66	64	12.6	12.2
P29	Extruded	24.2	54.9	1.3	3.9	3.2	7.0	63	72	14.9	17.1
P30	Flakes	0.7	67.1	3.1	9.3	2.2	11.5	62	62	12.6	12.6
P31	Extruded	26.9	44.7	2.0	5.9	4.9	9.0	61	68	13.1	14.6
P32	Extruded	4.7	62.7	2.1	6.4	7.5	11.1	61	71	12.3	14.3
P33	Muesli	14.9	47.3	2.6	7.8	6.7	11.9	60	61	11.2	11.3
P34	Muesli	11.4	45.5	2.0	6.1	11.1	11.8	59	58	10.1	9.9
P35	Muesli	14.9	44.1	2.2	6.5	9.5	10.6	59	59	10.4	10.5
P36	Granola	22.3	38.9	1.4	4.2	13.7	14.4	59	53	10.8	9.8
P37	Granola	25.5	43.3	1.6	4.7	9.8	10.0	59	58	12.2	12.0
P38	Granola	22.3	41.6	1.9	5.8	10.5	11.6	55	54	10.5	10.4
P39	Granola	18.7	41.8	1.9	5.8	12.4	13.0	54	52	9.8	9.5
P40	Granola	18.5	41.7	1.9	5.7	12.8	13.1	52	52	9.4	9.4
P41	Granola	22.1	41.4	1.9	5.7	10.3	12.0	52	54	9.9	10.3
P42	Granola	22.2	42.3	1.8	5.4	9.8	13.5	50	56	9.7	10.8
Min		0.7	33.4	0.7	2.0	1.2	4.9	50	52	9.4	9.4
Max		30.0	74.7	3.2	9.7	13.7	14.4	83	82	20.4	20.2
Mean		20.1	51.1	1.7	5.2	5.9	9.0	68	68	14.7	14.7
SD		8.5	10.0	0.6	1.7	3.8	2.5	9.2	8.2	3.1	2.9

**Table 2:** Glycemic index (GI_i_) and correcting factor (a_i_) of common glycemic carbohydrates, including glycemic sugar alcohols.

Glycemic Carbohydrates		GI_i_	a_i_
Monosaccharides	Glucose	100	1
	Fructose	20	1
	Galactose	20	1
Disaccharides	Maltose	105	1
	Trehalose	70	1
	Sucrose	62	1
	Lactose	47	1
	Isomaltulose	32	1
Polysaccharides	Maltotriose	110	1
	Maltotetraose	110	1
	Starch	110	%RDS/100
	Maltodextrin	110	1
Sugar alcohols	Maltitol	35	1
	Xylitol	12	1

**Table 3:** GI-lowering power (b_i_) of common macronutrient as defined by empirical data fitting for starch-based products.

GI-lowering Macronutrients		b_i_
Carbohydrates	β-glucan	0.6
	Fiber soluble	0.3
	Fiber insoluble	0.1
Others	Fat	0.6
	Protein	0.6
	Ashes	0.1
	Water	0.0
